# Editorial: Depression across cultures and linguistic identities

**DOI:** 10.3389/fpsyg.2024.1538489

**Published:** 2024-12-20

**Authors:** Katarzyna Milana Broczek, Marie-Christine Gely-Nargeot, Pietro Gareri

**Affiliations:** ^1^Polish Society of Gerontology, Warsaw, Poland; ^2^Department of Psychology, Université Paul Valéry, Montpellier III, Montpellier, France; ^3^Center for Cognitive Impairment and Dementia, Catanzaro Lido - Unit of Frailty Care, Azienda Sanitaria Provinciale Catanzaro, Dipartimento delle Cure Primarie Catanzaro, Catanzaro, Italy

**Keywords:** depressive symptoms, cultural differences, language, generational differences, anxiety

Depression is not only considered the most prevalent mental health problem worldwide, but it is also a common term used in everyday life, personal conversations, newspapers, and broad range of mass media. The Internet is full of descriptions by the icons of film, literature, and sports suffering and recovering from depressive symptoms. If depression affects individuals across diverse cultures and linguistic identities, the perception, expression, and treatment of depression can nonetheless differ significantly between cultures and languages. And there is a pending question how heterogenous faces of depression are understood in societies with different cultural backgrounds and speaking different languages. In fact, the way depression is perceived is deeply influenced by cultural values and norms: collectivist societies may view depression as a failure of group integration or family harmony, while individualist cultures might see it as a personal struggle. Some cultures stigmatize mental health conditions more heavily, leading individuals to suppress symptoms or interpret them differently. This affects both self-perception and how others in a given society recognize and respond to depressive symptoms.

The perception of self and self-description seem important factors in mental health and, specifically, depression. According to one of many theories of depression, self-focused attention is one of the main features of depressive symptomatology and, therefore, the use of first-person pronouns is marked in depressed individuals. The pronoun “I” is always situated in a social context, influenced by individualistic values, whether it means loneliness or being in a family or a community. These cultures, like western one, often emphasize personal narratives, leading to a tendency to discuss emotional states explicitly, both in personal conversations and clinical settings. In Asian societies, where collectivist values are highly prioritized, emotional struggles are more likely to be internalized or expressed indirectly. In consequence, differences in cultural backgrounds are very likely to influence the self-perception in healthy and depressed individuals.

The criteria for diagnosing depressive disorders are explicitly described by DSM-5 (DSM-5) (American Psychiatric Association, 2022), translated to many languages and introduced in various countries. One of the main criteria of depressive disorders is low mood including feeling sad, empty or hopeless. Such feelings may be shared by all human beings, but the expression of sadness, emptiness, and hopelessness may differ substantially between societies with certain traditions of self-exposure and level of openness in communication. Moreover, translations of the words describing symptoms of depression may differ in shades of meaning.

These different terms carry subtle connotations which might influence how individuals describe and perceive their emotional state. For example, the word sadness might be substituted in English by: grief, heartache, dolefulness, sorrow, regret, gloom, unhappiness, while in Spanish it encompasses tristeza, dolor, and pena to mention just a few. Additionally, generational changes in language may contribute to different patterns of depressive symptoms between the younger and older generations. In this way, generational differences add another layer of complexity. For example, younger generations in many globalized societies, exposed to Western media and mental health awareness campaigns, might adopt terms like “depression” or “burnout,” while older generations might use more culturally traditional expressions, such as “feeling heavy-hearted” or “having a dark spirit.” Depressed older adults may not feel “sad.” They may complain, instead, of physical complaints, such as arthritis pain, which is often the predominant symptom of depression in the elderly (Yuan et al., 2024).

The diagnosis of depression as any other mental health problem is based on the relationship between patients and clinicians. This variability underscores the importance of culturally sensitive approaches in diagnosing and treating depression. Clinicians and researchers must consider not only the linguistic and cultural contexts of their patients, but also how these factors influence the experience and communication of depressive symptoms. For example, the assessment of depressive symptoms may be difficult in indigenous people, migrants, and refugees representing different beliefs and values than the evaluating clinicians. Proper validation of screening tools for the assessment of psychological status is of great importance (Meldrum et al., 2023).

The present Research Topic includes four articles dedicated to the relationship between symptoms of depression, cultural background and linguistic aspects.

The work of Meneguzzo et al. described body self-evaluation in people with and without mood disorders living in Italy. Individuals with mood disorders tended to overestimate their body weight, but other descriptors of body perception were interestingly similar between the two groups.

Trifu et al. attempted to characterize linguistic markers of depression in patients with major depressive disorder (MDD) undergoing treatment in inpatient or outpatient setting in Romania. Automated language analysis revealed that MDD patients had different sentence structures than people without depression. Interestingly, first person pronoun was used more often in its plural than singular form.

The work of Miao is dedicated to tracing the history and conceptual evolution of “anxiety” in Chinese including linguistic aspects. The author provided captivating examples from historical medical lexicons.

Yamashita et al. presented a very interesting view of the psychological situation of technical interns from Vietnam living in Japan in relationship to their financial status.

The above four articles included in the Research Topic encompass a broad context of linguistic determinants of depressive disorders. Including cultural and, specifically, linguistic perspective into the research on mental health facilitates better understanding of the symptomatology and indicates the need to focus attention on multicomponent cultural bases of mental health.
